# Population Genetics and Antimicrobial Susceptibility of Canine *Campylobacter* Isolates Collected before and after a Raw Feeding Experiment

**DOI:** 10.1371/journal.pone.0132660

**Published:** 2015-07-14

**Authors:** Satu Olkkola, Sara Kovanen, Johanna Roine, Marja-Liisa Hänninen, Anna Hielm-Björkman, Rauni Kivistö

**Affiliations:** 1 Department of Food Hygiene and Environmental Health, Faculty of Veterinary Medicine, University of Helsinki, Helsinki, Finland; 2 Department of Equine and Small Animal Medicine, Faculty of Veterinary Medicine, University of Helsinki, Helsinki, Finland; Cornell University, UNITED STATES

## Abstract

In recent years, increasing numbers of consumers have become interested in feeding raw food for their pet dogs as opposed to commercial dry food, in the belief of health advantages. However, raw meat and internal organs, possibly contaminated by pathogens such as *Campylobacter* spp., may pose a risk of transmission of zoonoses to the pet owners. *Campylobacter jejuni* is the leading cause of bacterial gastroenteritis in humans but *C*. *upsaliensis* has also been associated with human disease. In this study we investigated the effect of different feeding strategies on the prevalence of *Campylobacter* spp. in Finnish dogs. We further characterized the isolates using multilocus sequence typing (MLST), whole-genome (wg) MLST and antimicrobial susceptibility testing. Dogs were sampled before and after a feeding period consisting of commercial raw feed or dry pellet feed. Altogether 56% (20/36) of the dogs yielded at least one *Campylobacter*-positive fecal sample. *C*. *upsaliensis* was the major species detected from 39% of the dogs before and 30% after the feeding period. Two *C*. *jejuni* isolates were recovered, both from raw-fed dogs after the dietary regimen. The isolates represented the same genotype (ST-1326), suggesting a common infection source. However, no statistically significant correlation was found between the feeding strategies and *Campylobacter* spp. carriage. The global genealogy of MLST types of dog and human *C*. *upsaliensis* isolates revealed weakly clonal population structure as most STs were widely dispersed. Major antimicrobial resistance among *C*. *upsaliensis* isolates was against streptomycin (STR MIC > 4mg/l). Apart from that, all isolates were highly susceptible against the antimicrobials tested. Mutations were found in the genes *rpsL* or *rpsL* and *rsmG* in streptomycin resistant isolates. In conclusion, increasing trend to feed dogs with raw meat warrants more studies to evaluate the risk associated with raw feeding of pets in transmission of zoonoses to humans.

## Introduction

Campylobacteriosis is the most common bacterial gastrointestinal disease in humans worldwide. The major species causing human disease are *Campylobacter jejuni* and *Campylobacter coli*. However, *Campylobacter upsaliensis*, often isolated from dogs [1–3] has been described as a cause of human disease including gastroenteritis and bacteremia [4–6] and dog ownership or contact with dogs has been identified as a risk factor for human campylobacter infections [7,8]. In dogs, asymptomatic carriage is common and especially *C*. *upsaliensis* is frequently isolated from both symptomatic and asymptomatic dogs [9,10]. However, it has been suggested that *C*. *jejuni*, and especially certain genotypes (i.e. ST-45), are significantly more prevalent among diarrheic than non-diarrheic dogs [11]. Furthermore, Chaban et al. (2010) showed that diarrheic dogs were more likely to shed *Campylobacter* spp., *C*. *jejuni* and *C*. *coli* among others, at significantly higher concentrations compared to healthy dogs [12].

In a previous study, genotyping by randomly amplified polymorphic DNA typing could not distinguish between two human clinical *C*. *jejuni* isolates and four canine strains suggesting that dogs are significant reservoirs of *Campylobacter* and contribute to human enteric infections [13]. Multilocus sequence typing (MLST) has been a valuable method in studies of molecular epidemiology, population structure and source attribution of *Campylobacter* [14–16]. However, little data on the genetic diversity of *C*. *jejuni* [8,11,17] and especially *C*. *upsaliensis* isolates in dogs worldwide exist [18].

In recent years, a growing number of consumers have become interested in offering raw meat-based feed for their dogs, considered as more natural and healthy as opposed to commercial dry food (http://www.barfaustralia.com). Raw dog food typically includes uncooked meat, edible bones and internal organs, such as liver, from various animals, for example chickens, bovines and pigs. In addition, Biologically Appropriate Raw Food or Bones and Raw Food (BARF) typically contains at least fruit and vegetables, and also possibly eggs and dairy products [19].

Campylobacters are commonly isolated from raw chicken meat [20] and also from bovine, pig and chicken livers [21–23]. In a recent Canadian study, DNA-based methods further revealed the presence of various *Campylobacter* spp. in ground beef, used also as dog raw food, including *C*. *jejuni* (3.9%) and *C*. *upsaliensis* (2.9%) [24]. However, in two previous studies, no *Campylobacter* spp. were found when evaluating the bacteriological quality of commercial raw canine diets [25] or raw meat diets [26]. Further, the only study concerning canine raw feeding found one of the 42 (2.6%) raw meat-fed dogs and none of the 49 control dogs to be positive for *C*. *jejuni* [27].

Campylobacteriosis in humans is usually self-limiting but severe cases or immunocompromised patients are treated with antimicrobials, preferentially with macrolides or fluoroquinolones [28]. Intravenous aminoglycosides can also be used in serious campylobacter bacteremia [29]. Antimicrobial resistance of *C*. *jejuni* from dogs (and cats) has been evaluated in some studies with resistance rates varying between 0–60% for quinolones, 0–40% for tetracycline and 0–12% for erythromycin with lowest prevalence of resistant isolates found in Norway [10,11,30,31]. However, only a limited number of studies on the prevalence or mechanisms of antimicrobial resistance of canine *C*. *upsaliensis* strains exist [10,31,32]. Interestingly, in a Norwegian study, most canine *C*. *upsaliensis* isolates were resistant to streptomycin and one strain was also resistant to nalidixic acid [31] and all outbreak-associated *C*. *upsaliensis* isolates from children in day care centers in Brussels were also resistant to streptomycin [5]. Another study found that *C*. *upsaliensis* strain RM3195 isolated from a human patient was resistant to nalidixic acid, oxytetracycline and novobiocin but not to streptomycin or most of the β-lactam antibiotics. However, the authors found no known mutations, which could explain the quinolone resistance [33].

In this study our aims were i) to investigate the prevalence of *Campylobacter* spp. in dogs with reference to the feeding strategy before and after the feeding regimen, consisting of either raw or dry commercial dog food, (ii) to analyse the MLST and wgMLST (whole-genome MLST) types of *Campylobacter* spp. isolates in local and global view and iii) to study the antimicrobial resistance patterns and mechanisms of the canine *Campylobacter* isolates.

## Materials and Methods

### Sample collection

Altogether, 36 Staffordshire bull terriers originating from a total of 30 households in Southern Finland, either healthy or diagnosed with atopic dermatitis, were included in this study. The dogs were divided in two groups with 15 dogs receiving commercial dry pellet feed and 18 dogs receiving raw feed consisting of meat, bones and organs from pork, chicken and lamb and/or beef, turkey and salmon. The feeding period lasted for 4 to 5 months. Owners of three dogs did not keep to the feeding regimen and those dogs were excluded from the *Campylobacter* prevalence analysis. Fecal samples were collected twice, before and after the feeding period, by the owners as a three-day pooled sample and kept refrigerated prior to analysis performed within 0 to 3 days from collection. All animal work has been conducted according to relevant national and international guidelines and with the dog owners' consent. This study was authorized by a written permission from the National Animal Experiment Board (Eläinkoelautakunta ELLA, decision number ESAVI/3244/04.10.07/2013) under Regional State Administrative Agency for Southern Finland.

### Bacterial isolates

Fecal samples were suspended in 0.9% saline (1g feces/1ml saline) and a 10 μl loopful of this suspension was plated on modified charcoal cefoperazone deoxycholate agar (mCCDA) (CM739, Oxoid Ltd., Basingstoke, Hampshire, UK) with the selective supplement (SR155, Oxoid Ltd.) and incubated for up to 7 days at 37°C in jars (MART, anoxomat, Netherlands) in microaerobic conditions (6% O_2_, 10% CO_2_, 5% H_2_). At first, samples were also enriched in Bolton broth (Oxoid), with 5% horse blood and selective supplement (SR183E, Oxoid Ltd.), and incubated microaerobically at 37°C for 48 h but since all enriched samples, unlike direct culture, were consistently negative for *Campylobacter* spp. we ceased using this method. Colonies showing typical growth on mCCDA and morphology in gram stain were confirmed as *Campylobacter* spp. using genus specific PCR [34] and as *C*. *jejuni* or *C*. *upsaliensis* with species specific PCR [34,35]. Bacterial isolates were identified at baseline with the individual dog numbers (DRXX) and after the feeding period with number two at the end (DRXX_2).

### Whole genome sequencing, MLST and data analysis

Draft genome sequences of all the recovered *C*. *jejuni* and *C*. *upsaliensis* isolates were determined using Illumina MiSeq or HiSeq technology (Nextera library, Nextera XT paired end kit, 250 cycles). NGS library preparation, enrichment and sequencing were performed by the Institute for Molecular Medicine Finland (FIMM Technology Centre, University of Helsinki, Finland). The paired-end reads were assembled into contigs using SPAdes 3.1.1 [36]. MLST types were assigned using the *Campylobacter* MLST database (pubMLST.org/campylobacter/) and new allele sequences were submitted to the non *jejuni/coli Campylobacter* MLST database (http://pubmlst.org/campylobacter/).

The draft genomes were further analysed for whole-genome MLST using Genome profiler (GeP) [37]. The genomes were annotated in RAST (Rapid annotation using subsystem technology) and the resulting gbk files were used, as suitable, as reference genomes in the GeP analysis [37]. The NeighborNet networks, representing allelic distance matrix of the shared loci of the isolates, were constructed using SplitsTree4 [38] and edited using CorelDRAW X6.

The software ClonalFrame ver. 1.2 [39] was used to generate a genealogy tree of all known *C*. *upsaliensis* STs from the MLST database (http://pubmlst.org/campylobacter/) based on the sequences of the seven housekeeping genes with 50 000 iterations, 50 000 burn-in iterations and every 100^th^ three was sampled.

A full minimum spanning tree of all MLST profiles present in the PubMLST database and isolate data (origins, i.e. country and source combinations with more than 2 isolates were included) from this study as well as those present in the PubMLST database and published by Parsons et al. (2012) was generated using the goeBURST algorithm [40,41] and visualized using PHYLOViZ 1.1 [42].

The nucleotide sequences of the *C*. *upsaliensis* genes *gyrA*, *rsmG*, *rpsL* and *rrs* were searched by BLAST in RAST, translated using EMBOSS Transeq (http://www.ebi.ac.uk/Tools/st/emboss_transeq/) and aligned for comparison using MUSCLE (http://www.ebi.ac.uk/Tools/msa/muscle/). The sequences were compared to those of *C*. *upsaliensis* RM3195.

### MIC determination

All the recovered *C*. *jejuni* (n = 2) and *C*. *upsaliensis* (n = 24) isolates were screened for antimicrobial resistance for erythromycin (ERY), tetracycline (TET), streptomycin (STR), gentamicin (GEN) and for the quinolones ciprofloxacin (CIP) and nalidixic acid (NAL) with the broth microdilution method (VetMIC Camp, National Veterinary Institute, Uppsala, Sweden) according to the manufacturer’s instructions. However, due to the fastidious nature of some of the *C*. *upsaliensis* isolates, a modified method utilizing Nutrient broth (Oxoid Ltd., Basingstoke, Hampshire, UK) with 5% blood (Labema, Kerava, Finland) instead of cation-adjusted Muller-Hinton broth (Difco, Becton-Dickinson and Company, Sparks, USA) with 5% blood was used for part of the *C*. *upsaliensis* isolates [43]. The agar dilution method (Clinical and Laboratory Standards Institute M31-A3) was used to determine and confirm the STR resistance levels of *C*. *upsaliensis* isolates. The epidemiological cut-off values (ECOFFs) for *C*. *jejuni*, as determined by the European Committee on Antimicrobial Susceptibility Testing (EUCAST, www.eucast.org), were applied to distinguish between wild type (also referred to as susceptible) and non-wild type (also referred to as resistant) populations and were as follows: MIC > 4 mg/l for ERY and STR, MIC > 0.5 mg/l for CIP, MIC > 1 mg/l for TET, MIC > 2 mg/l for GEN and MIC > 16 mg/l for NAL. Due to the lack of data concerning MIC distributions of *C*. *upsaliensis*, these ECOFFs were also applied for it.

## Results

### Occurrence and MLST types of *Campylobacter* spp. before and after raw feeding

The main results are presented in Table 1. A total of two *C*. *jejuni* and 24 *C*. *upsaliensis* isolates were detected from the feces of the 36 dogs included in this study. Altogether 20 dogs (55.6%) were positive for *Campylobacter* spp. in at least one sampling and six (16.7%) were positive in both samplings. Of the 33 dogs that kept to the feeding regimen, at baseline 13 (39.4%) were positive for *C*. *upsaliensis* and after the feeding period two dogs (6.1%) carried *C*. *jejuni* and 10 dogs (30.3%) *C*. *upsaliensis*. The *C*. *jejuni* isolates were recovered from two raw-fed dogs from different households living approximately 10 kilometers apart in the Helsinki area. Both dogs were *Campylobacter*-negative at the beginning (Table 1). Four new *C*. *upsaliensis*-positive dogs appeared after the feeding regimen; however, seven positive dogs at baseline seemed to have cleared off their *C*. *upsaliensis* carriage. No statistically significant association was found between raw or dry pellet feed diet and prevalence of *C*. *jejuni* or *C*. *upsaliensis*.

**Table 1:** Feeding strategies and *Campylobacter* status of studied dogs at baseline sampling and after the feeding regimen.

		Baseline sampling				Sampling after the feeding regimen		
Dog^1^	Age (y)	Campylobacter status	ST	Resistance^3^	Feeding strategy^2^	Campylobacter status	ST	Resistance^3^
DR2S	6	neg.	-	-	D	neg.	-	-
DR8S	6	neg.	-	-	D	neg.	-	-
DR9S	9	*C*. *upsaliensis*	ST-158	**STR**	R	*C*. *upsaliensis*	ST-159	STR
DR10S^a^	5	neg.	-	-	R	*C*. *jejuni*	ST-1326	S
DR11S^a^	7	neg.	-	-	R	neg.	-	-
DR15S^b^	6	*C*. *upsaliensis*	ST-160	STR	R	*C*. *upsaliensis*	ST-160	STR
DR18S^b^	4	*C*. *upsaliensis*	ST-160	STR	R	*C*. *upsaliensis*	ST-160	STR
DR22S	6	*C*. *upsaliensis*	ST-165	CIP-NAL-**STR**	R	neg.	-	-
DR24S	3	neg.	-	-	D	neg.	-	-
DR25S	12	neg.	-	-	V	neg.	-	-
DR26S	3	neg.	-	-	R	neg.	-	-
DR27S	10	neg.	-	-	D	neg.	-	-
DR28S	1	*C*. *upsaliensis*	ST-166	S	R	neg.	-	-
DR31S	3	neg.	-	-	D	neg.	-	-
DR35S	5	neg.	-	-	R	neg.	-	-
DR36S	10	neg.	-	-	R	*C*. *upsaliensis*	ST-167	**STR**
DR37S	5	neg.	-	-	D	neg.	-	-
DR39S	3	*C*. *upsaliensis*	ST-169	S	D	neg.	-	-
DR40S	1	neg.	-	-	D	*C*. *upsaliensis*	ST-170	**STR**
DR41S	3	neg.	-	-	R	*C*. *jejuni*	ST-1326	S
DR42S	1	*C*. *upsaliensis*	ST-171	STR	R	neg.	-	-
DR43S	2	neg.	-	-	V	neg.	-	-
DR44S	3	*C*. *upsaliensis*	ST-172	STR	D	*C*. *upsaliensis*	ST-172	STR
DR45S	6	neg.	-	-	V	*C*. *upsaliensis*	ST-174	STR
DR46S^c^	7	neg.	-	-	D	neg.	-	-
DR47S^c^	7	*C*. *upsaliensis*	ST-166	S	D	neg.	-	-
DR48S	3	*C*. *upsaliensis*	ST-176	STR	R	neg.	-	-
DR49S	4	neg.	-	-	D	neg.	-	-
DR50S	5	neg.	-	-	R	neg.	-	-
DR51S	9	neg.	-	-	R	neg.	-	-
DR52S^d^	4	neg.	-	-	R	neg.	-	-
DR53S^d^	1	neg.	-	-	R	*C*. *upsaliensis*	ST-177	S
DR55S^e^	5	*C*. *upsaliensis*	ST-178	STR	D	*C*. *upsaliensis*	ST-178	STR
DR56S	3	*C*. *upsaliensis*	ST-167	**STR**	R	neg.	-	-
DR59S^e^	6	*C*. *upsaliensis*	ST-181	S	D	*C*. *upsaliensis*	ST-182	**STR**
DR60S^e^	4	neg.	-	-	D	*C*. *upsaliensis*	ST-182	**STR**

^1^ Dogs coming from the same household are indicated with the same superscript letter a-e.

^2^ R, raw; D, dry; V, varied (did not follow the feeding regimen).

^3^ S, susceptible; CIP, ciprofloxacin; NAL, nalidixic acid; STR, streptomycin (bolded when STR MIC > 512 mg/l).

Both *C*. *jejuni* isolates represented sequence type (ST) 1326 and the 24 *C*. *upsaliensis* isolates were assigned to 16 STs, all of which were novel i.e. not existing in the PubMLST database (Table 1). Four of the six dogs that were *C*. *upsaliensis*-positive in both samplings yielded the same ST both times. The remaining two dogs had *C*. *upsaliensis* isolates with completely different allelic profiles (DR9S, ST-158 and ST-159, Table 1) or shared sequences in only two MLST loci (DR59S, ST-181 and ST-182, Table 1) in the successive samplings.

### Whole-genome multilocus sequence typing (wgMLST)

**Fig 1:**
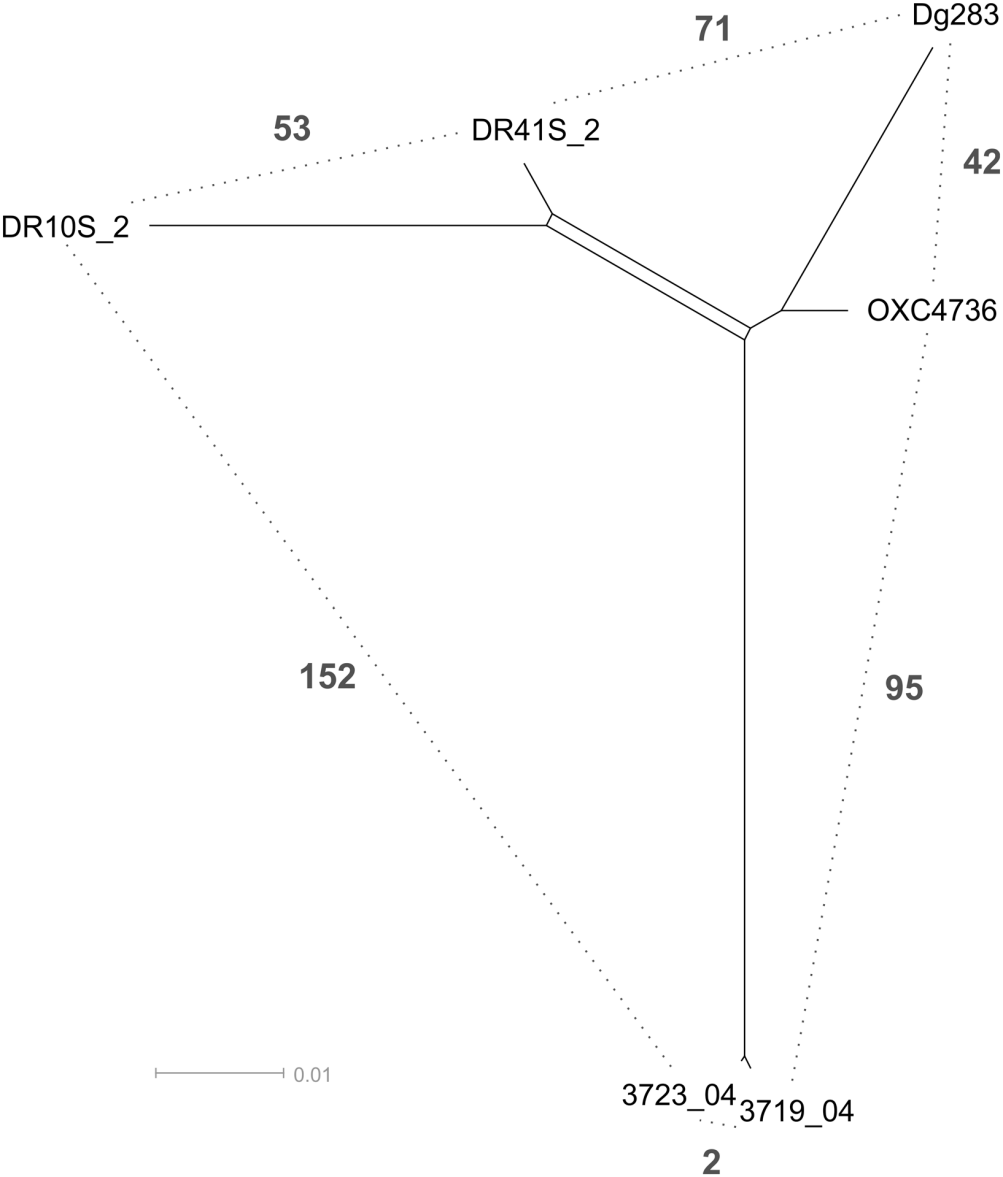
wgMLST of *C*. *jejuni* ST-1326 isolates. SplitsTree of the NeighborNet network (1,457 shared genes) of *C*. *jejuni* ST-1326 isolates using GeP (Zhang et al., 2015). Dashed lines and numbers indicate the number of allelic differences observed between the pair of isolates. Two UK isolates (OXC4736 and Dg283) obtained from PubMLST isolate database (PubMLST id 18439 and 25960) and two chicken isolates (3719_04 and 3723_04) from our own collection (Llarena et al. 2015) were included as reference strains.

All available draft genomes representing *C*. *jejuni* ST-1326 from the PubMLST database (pubMLST.org/camplylobacter) and from our own collection were included in the wgMLST analysis, in addition to the canine ST-1326 isolates identified in the present study (Fig 1). Among the 1,457 shared genes between all the genomes, the least number of allelic differences (2) was seen between two Finnish chicken isolates (3719_04 and 3723_04, Fig 1), detected on the same day from different slaughter batches reared at the same farm. However, our dog isolates were more similar to each other with 53 allelic differences (DR10S_2 and DR41S_2, Fig 1), and to the two UK isolates with allelic differences ranging from 44 (DR41S_2 and OXC4736, human stool isolate) to 111 (DR10S_2 versus Dg283, isolate from an undefined animal) than to the Finnish chicken isolates.

**Fig 2:**
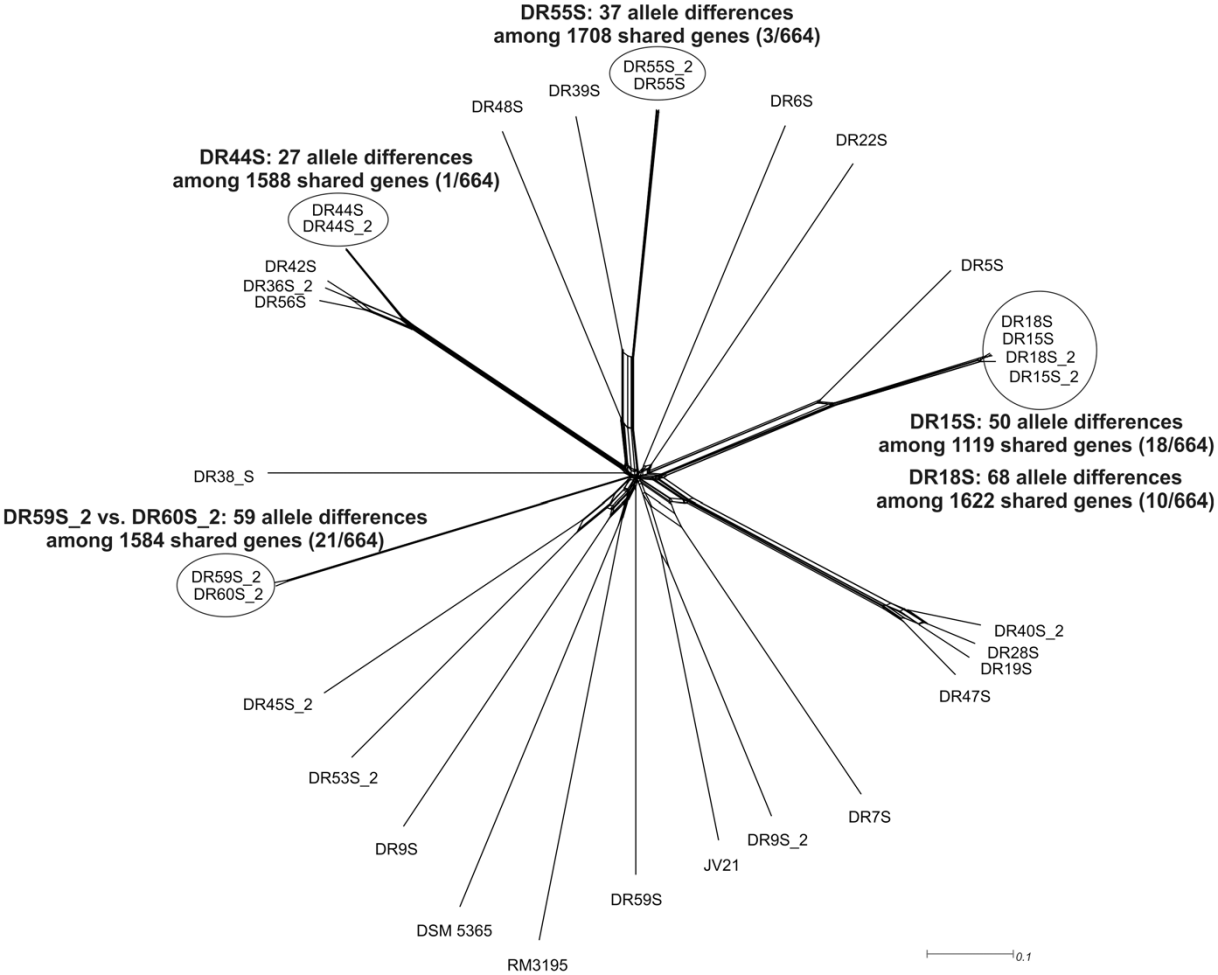
wgMLST of *C*. *upsaliensis* isolates. SplitsTree of the NeighborNet network (664 shared genes) of all available *C*. *upsaliensis* whole genomes, including three reference strains and isolates from dogs that were sampled only once and thus not included in this study, using GeP (Zhang et al., 2015). A new GeP analysis was performed for all closely related isolates and the results are shown next to the pair of isolates. The number of allelic differences, observed in the primary GeP analysis among the 664 shared genes, are shown in parenthesis. The reference genomes DSM 5365, JV21 and RM3195 were obtained from GenBank (accession numbers JHZN00000000, NZ_AEPU00000000 and NZ_AAFJ00000000).

The NeighborNet network of the wgMLST analysis of the Finnish canine *C*. *upsaliensis* isolates is shown in Fig 2. Altogether 664 genes were shared between the genomes. Lowest numbers of allelic differences at the wgMLST level were seen among the isolates from the same individual dogs representing the same STs, collected before and after the feeding period, ranging from 1 (DR44S, Fig 2) to 18 (DR15S, Fig 2). Furthermore, isolates collected from two different dogs living in the same household clustered closely together showing only 10 to 23 (DR15S and DR18S, Fig 2) and 21 (DR59S_2 and DR60S_2, Fig 2) allelic differences among the 664 shared genes. In addition, six isolates from the same number of dogs, originating from different households, formed two clusters, however, showing relatively high numbers of allelic differences ranging from 49 (DR36S_2 versus DR42S, Fig 2) to 120 (DR28S versus DR47S, Fig 2), while rest of the isolates showed much higher genetic diversity.

### Global genealogy of *C*. *upsaliensis* isolates from different sources

**Fig 3:**
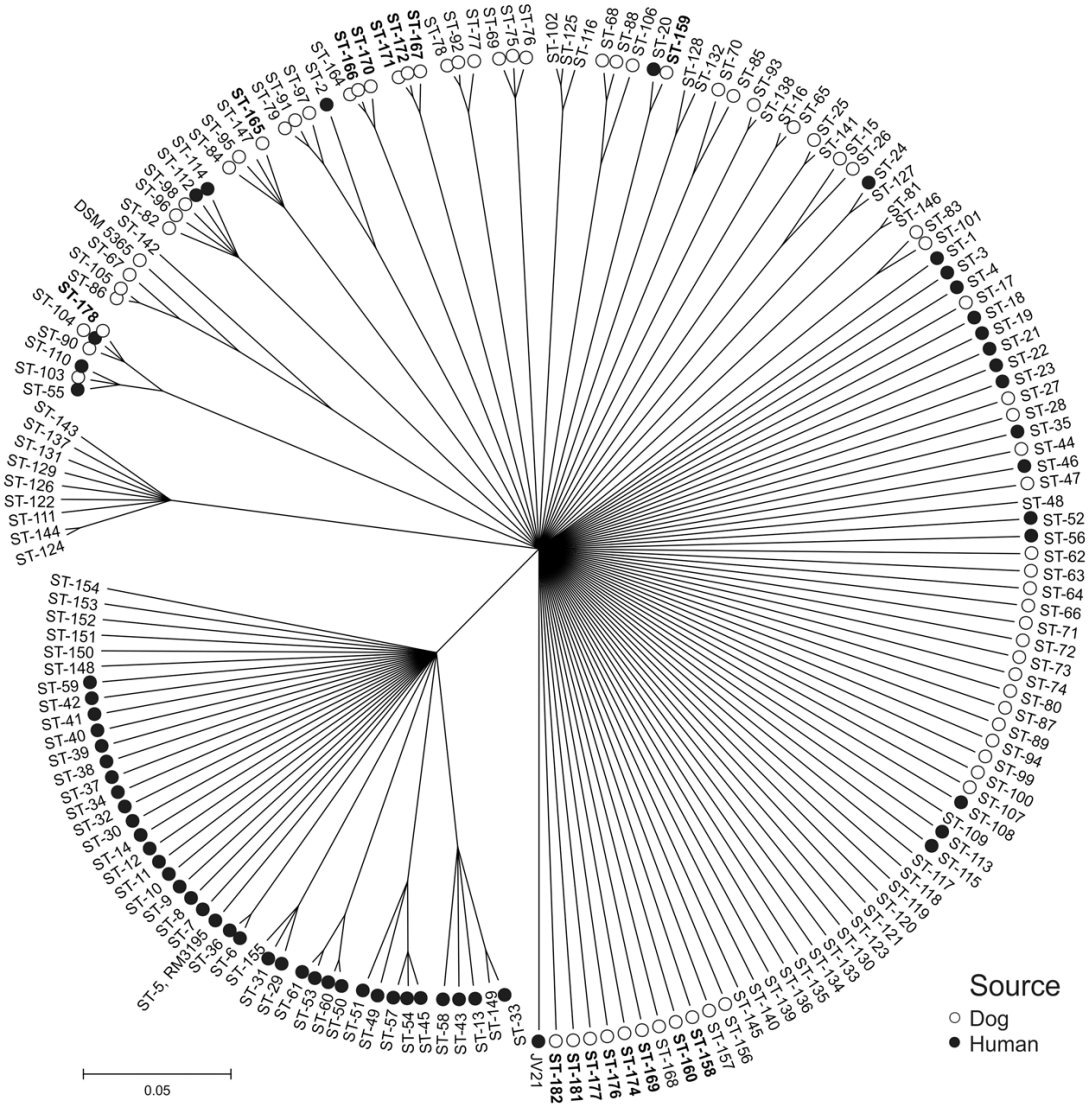
ClonalFrame genealogy tree based on all known *C*. *upsaliensis* MLST allele sequences. Novel STs reported in this study are indicated in bold. The sources of the isolates are indicated based on the information available in the PubMLST *Campylobacter* non *jejuni/coli* database and in Parsons et al. (2012).

Similar clusters that occurred among our isolates in the wgMLST NeighborNet network were also detected in the ClonalFrame genealogy tree, based on the distinct *C*. *upsaliensis* MLST allele sequences at each locus (ST-166 and ST-170 and ST-167, ST-171 and ST-172, Fig 3). One major clonal complex (ST-42 CC, Fig 3) formed a separate cluster including only human patient isolates (mainly gastroenteritis except the isolate RM3195 from a GBS patient). In addition, some small groups, representing isolates from both dogs (from this and previous studies) and human patients (previous studies) occurred as well as a bigger cluster representing nine STs from unknown/unpublished sources (Fig 3). Otherwise most of the STs showed only little phylogenetic relatedness and at least one third of the isolates seemed quite unrelated to each other, including eight (50%) of the *C*. *upsaliensis* STs of the present study.

**Fig 4:**
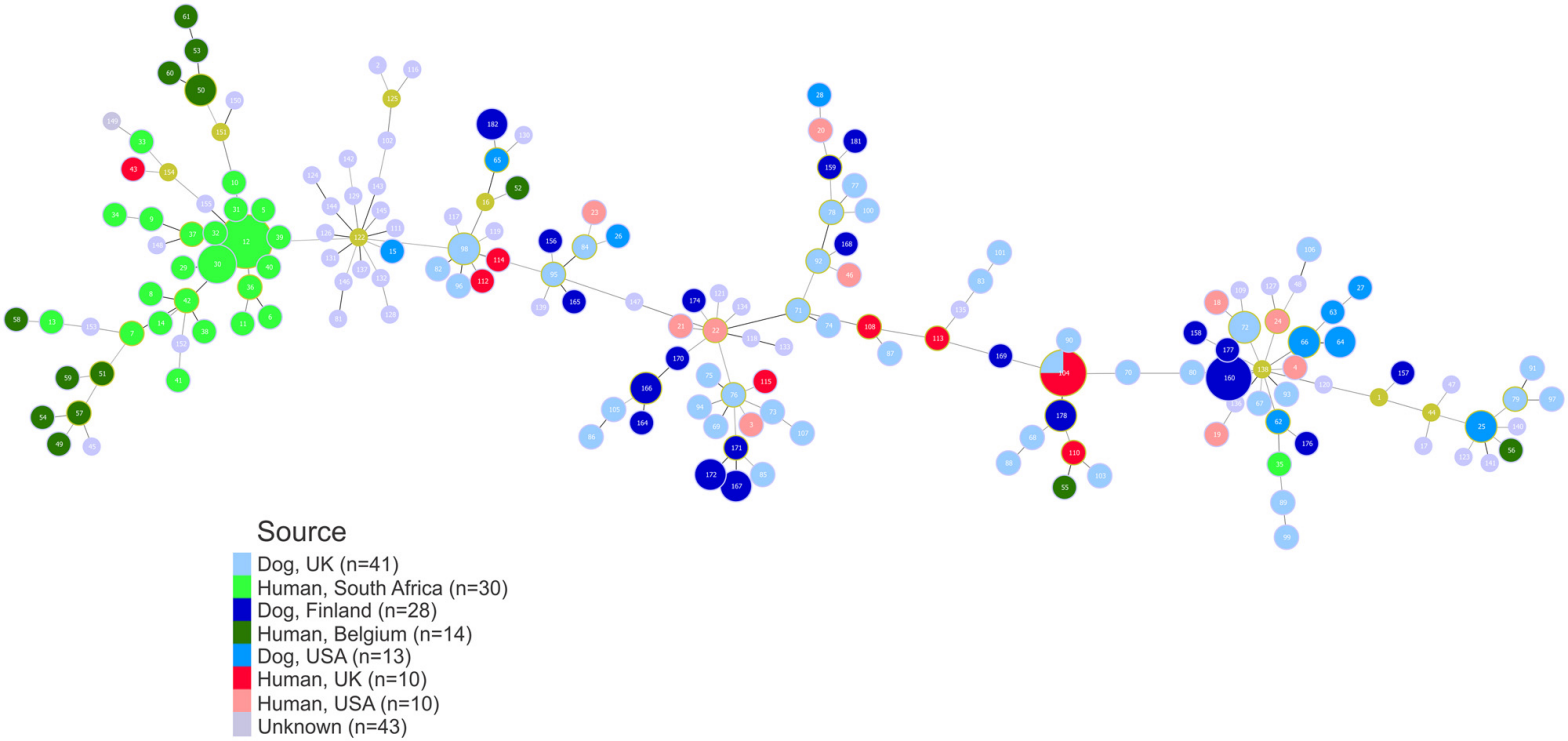
goeBURST full Minimum Spanning Tree (MST) of *C*. *upsaliensis* ST allelic profiles. Full MST of all *C*. *upsaliensis* allelic profiles present in PubMLST database overlaid by the isolation data (source and country combinations with more than 2 isolates were included), was generated using goeBURST and visualized with PHYLOViZ 1.1. The node sizes vary linearly with the number of isolates of a given ST. The links are color-coded for the number of differences i.e. darker links represent less allelic differences between the profiles than lighter links. Data used to create this figure is presented in S1 and S2 Tables.

The evolutionary descents of *C*. *upsaliensis* isolates were further inferred using the goeBURST algorithm implemented in PHYLOViZ and visualized as an extended full Minimum Spanning Tree (MST) (Fig 4), overlaid by data representing the sources of the isolates. Although the most single locus variants originated from the same source and country combination, some were also found among canine and human isolates originating either from the same or two different countries. Furthermore, similarly as the human and dog *C*. *upsaliensis* isolates from the UK and USA, the Finnish dog isolates were distributed throughout the phylogenetic tree. The only exception was the ST-42 CC cluster, wherein most of the isolates originated from South Africa and Belgium solely from humans and the cluster representing isolates from undefined sources (Fig 4), which was also identified in ClonalFrame analysis (Fig 3).

### Antimicrobial resistance of *Campylobacter* spp. isolates

The two *C*. *jejuni* isolates were susceptible to all the antimicrobials studied. Among *C*. *upsaliensis*, the most notable resistance trait was resistance to streptomycin with 79% (19/24) of the isolates having MICs of >4 mg/l and with seven of these with MICs of >512 mg/l. The remaining five isolates had streptomycin MICs of 0.5–2 mg/l (S3 Table). All isolates with streptomycin MIC > 4 mg/l encoded arginine (AGA) in codon 88 of *rpsL*, while all the five susceptible isolates encoded lysine (AAA) in the same position (S3 Table). Further, all isolates with streptomycin MIC > 512 mg/l had various deletion or insertion mutations in *rsmG* leading to frameshift and a premature stop codon immediately downstream of the mutation site resulting in termination at amino acid number 13, 49, 137, 144 or 155 of the encoded 7-methylguanosine methyltransferase. Also some intermediate-level resistant (MIC 16 mg/l) and susceptible isolates showed truncation of *rsmG* but only the last 3–5 amino acids were lost in these cases (S3 Table). No resistance associated mutations in the sequences of *rrs* were detected. One *C*. *upsaliensis* isolate was resistant to ciprofloxacin (MIC 1 mg/l) and nalidixic acid (MIC > 64 mg/l) and had point mutation C257T in *gyrA* resulting in amino acid substitution Thr-86-Met. No resistance to TET, ERY or GEN was detected.

In two cases *C*. *upsaliensis* isolates had closely related STs but differing MICs for streptomycin, and no clear pattern in the distribution of resistant or susceptible isolates was identified (Table 1). However, the *C*. *upsaliensis* isolates that originated from the same dog in the consecutive samplings and represented the same ST (DR15S and DR15S_2; DR18S and DR18S_2; DR44S and DR44S_2; DR55S and DR55S_2, Table 1) always had same streptomycin MICs.

## Discussion

Dogs are common pets especially in industrialized countries and approximately 50,000 new dogs are registered annually in Finland (www.kennelliitto.fi). Dogs carrying *Campylobacter* spp. in their intestines may pose a risk of human infection by direct or indirect contact with fecal material of the animals [8]. In the present study, *C*. *upsaliensis* was the most common *Campylobacter* spp. found from dogs and *C*. *jejuni* was detected only in few cases, which is in accordance with several previous publications [3,44]. However, some studies [11,45] have found *C*. *jejuni* as the main species in canines, which could be due to the differences in the studied dog populations or isolation protocols.

Similarly to the results of Lenz et al. (2009) also we detected a low proportion of *C*. *jejuni* among the raw-fed group after the feeding period. Both *C*. *jejuni* isolates represented the same, rarely detected ST-1326 (ST-45 CC) that has previously been isolated from human patients, bovines, chickens, barnacle geese, grey seal pups and environmental water samples [46,47]. Furthermore, an association between ST-1326 and pet dog colonization was identified in a previous study from the Netherlands [8]. Since both *C*. *jejuni* isolates were detected from dogs living in different households, they likely originated from a common source, possibly from raw food. However, due to the low number of samples obtained, raw feed was not analysed in this study. Also, the option that these dogs acquired *C*. *jejuni* ST-1326 from other, possibly environmental-associated sources cannot be excluded since previous studies on wgMLST of *C*. *jejuni* have revealed that only few genetic differences (single nucleotide polymorphisms, SNPs) may occur among temporally and genetically related isolates [48,49] and in this study a total of 53 allelic differences were observed between the two *C*. *jejuni* ST-1326 isolates obtained from two unrelated dogs.

Whole-genome MLST analysis between *C*. *upsaliensis* isolates obtained from individual dogs 4–5 months apart revealed also relatively high numbers of allelic differences (range 27–68) among the isolates representing the same STs. Unfortunately, no data of the effect of long-term host colonization on the genomic variation of *C*. *jejuni* or *C*. *upsaliensis* exist. Therefore, more research should be conducted to estimate the microevolution occurring in *Campylobacter* genomes during long-term colonization in different hosts.

The global genealogy of the MLST types of *C*. *upsaliensis* revealed a highly diverse population, in which *C*. *upsaliensis* isolates, detected from both dogs and human patients, were dispersed throughout the phylogenetic network. MLST types showed also some degree of overlap resulting in a hypothesis that in principle, all dog isolates could be capable of causing disease also in humans. More isolates from various sources are needed to better understand the genealogy of *C*. *upsaliensis* MLST types, as only two out of five countries had deposited MLST types from both humans and dogs and other sources were lacking altogether.

Macrolides and fluoroquinolones are the first and second choice antibiotics when antimicrobial treatment of human campylobacteriosis is warranted [50]. In Finland, there are no data available on the consumption of antimicrobial agents per animal species yet but there are several fluoroquinolone containing drugs registered for small animals. However, both *C*. *jejuni* isolates were susceptible and, apart from streptomycin, most *C*. *upsaliensis* isolates were also susceptible to all the antimicrobials studied. Quinolone resistance in a small percentage among *C*. *upsaliensis* from pets has been detected previously for example in Belgium, Italy and Norway [10,31,32]. Resistance mechanisms for quinolones in *C*. *jejuni* and *C*. *coli* are well described and resistance is mediated by single point mutation in the *gyrA* gene and also by the increased activity of the CmeABC efflux pump [51]. The most commonly described resistance conferring mutation is C257T in *gyrA*, leading to amino acid substitution Thr-86-Ile in *C*. *jejuni* and *C*. *coli* and resulting in high level of quinolone resistance, while other substitutions (Thr-86-Lys, Asp-90-Asn, Asp-90-Ala, Ala-70-Thr, Thr-86-Ala) have been associated with low level of quinolone resistance or resistance to nalidixic acid alone [51–53]. We describe here the same point mutation C257T in *C*. *upsaliensis*, but interestingly, this point mutation leads to Thr-86-Met substitution in this species leading to lower level of ciprofloxacin resistance (1 mg/l). To our knowledge, this is the first description of Thr-86-Met mutation in GyrA in connection to quinolone resistance.

Our finding that 79% of the *C*. *upsaliensis* isolates had increased MICs for streptomycin is in accordance with previous studies [5,31]. Streptomycin resistance in *C*. *jejuni* and *C*. *coli* can be conferred by enzymatic modification enzymes encoded in plasmids or chromosomally [54,55] and we have also shown that mutations in the *rpsL* gene codons 43 and 88 lead to streptomycin resistance in *C*. *coli* [56]. This latter resistance mechanism is quite well characterized also in other organisms, such as *E*. *coli*, *M*. *tuberculosis* and *Helicobacter pylori* [57–59]. Our finding that all *C*. *upsaliensis* isolates with streptomycin MIC > 4 mg/l encode arginine in codon 88 (and lysine in 43) of the *rpsL* gene is consistent with some former studies: mutations in codon 43 have been associated with a higher level of STR resistance, while those in codon 88 have resulted in more variable STR MICs [56,58,60]. In addition, various (often frameshift) mutations within *rsmG* (previously known as *gidB*) encoding 7-methylguanosine methyltransferase that methylates 16S rRNA, have been associated with low level of streptomycin resistance in a number of bacterial species and high frequency of emergence of streptomycin-resistant mutants. Furthermore, *rsmG rpsL* double mutants have been associated with a high-level streptomycin resistant phenotype in several bacterial species, such as *M*. *tuberculosis*, *Bacillus subtilis* and *E*. *coli* [61–63] and this was also observed in all highly resistant *C*. *upsaliensis* isolates described in this study.

## Conclusions

*C*. *upsaliensis*, showing a weakly clonal population structure, was the most common finding among dogs before and after the feeding regimen. No statistically significant correlation was found between the feeding strategies and the prevalence of *Campylobacter* spp. carriage. However, *C*. *jejuni* isolates with the same ST were recovered from two raw-fed dogs, suggesting a common source of infection. The main antimicrobial resistance detected among *C*. *upsaliensis* was against streptomycin and apart from that, the isolates were highly susceptible. Further studies should be conducted to reveal the significance of *C*. *upsaliensis* in human infections and to identify its sources and reservoirs worldwide. Also the role of raw-feeding versus direct transmission of *Campylobacter* species between different animals should be further investigated.

## Supporting Information

S1 Table *C*. *upsaliensis* STs and corresponding allele profiles used for Full MST (Fig 4). (XLSX)

S2 Table Epidemiological data associated with *C*. *upsaliensis* isolates used for Full MST image generated by PHYLOViZ (Fig 4). (XLSX)

S3 Table Streptomycin MICs and *rpsL* and *rsmG* mutations in spontaneous streptomycin resistant *C*. *upsaliensis* isolates. (XLSX)
